# Insomnia and paranoia

**DOI:** 10.1016/j.schres.2008.12.001

**Published:** 2009-03

**Authors:** Daniel Freeman, Katherine Pugh, Natasha Vorontsova, Laura Southgate

**Affiliations:** Department of Psychology, Institute of Psychiatry, King's College London, United Kingdom

**Keywords:** Delusions, Persecutory, Paranoia, Insomnia, Psychosis

## Abstract

Insomnia is a potential cause of anxiety, depression, and anomalies of experience; separate research has shown that anxiety, depression and anomalies of experience are predictors of paranoia. Thus insomnia may contribute to the formation and maintenance of persecutory ideation. The aim was to examine for the first time the association of insomnia symptoms and paranoia in the general population and the extent of insomnia in individuals with persecutory delusions attending psychiatric services. Assessments of insomnia, persecutory ideation, anxiety, and depression were completed by 300 individuals from the general population and 30 individuals with persecutory delusions and a diagnosis of non-affective psychosis. Insomnia symptoms were clearly associated with higher levels of persecutory ideation. Consistent with the theoretical understanding of paranoia, the association was partly explained by the presence of anxiety and depression. Moderate or severe insomnia was present in more than 50% of the delusions group. The study provides the first direct evidence that insomnia is common in individuals with high levels of paranoia. It is plausible that sleep difficulties contribute to the development of persecutory ideation. The intriguing implication is that insomnia interventions for this group could have the added benefit of lessening paranoia.

## 1 Introduction

Clinical experience indicates that many individuals with persecutory delusions have difficulties initiating and maintaining sleep. The extent of the problem has never been reported. Often the insomnia is simply a result of insufficient activity during the day and early retirement to bed. However the relationship between insomnia and paranoia may hold greater clinical and theoretical interest. The stressful experience of insomnia may lead to the lowering of mood and anomalies of experience that drive persecutory ideation. The intriguing clinical implication is that simple well-established interventions for sleep difficulties could lessen paranoid experience.

There is evidence consistent with the idea that sleep disturbance has a role in the development of paranoia. Insomnia is a risk factor for the development of emotional disorder (Ford and Kamerow, 1989; Breslau et al., 1996; Morphy et al., 2007) and an association with daytime mood disturbance has been repeatedly demonstrated (Riedel and Lichstein, 2000; Buysse et al., 2007). In separate research, paranoia has been strongly linked with negative affect (Freeman et al., 2008b; Bentall et al., in press), even being considered a type of anxious fear (Freeman and Freeman, 2008). Therefore insomnia may be one cause of the negative mood that leads to paranoia. Anomalies of experience such as perceptual distortions and hallucinations are also considered a key cause of paranoia (Maher, 1988; Freeman et al., 2008a); therefore it is germane that sleep deprivation has long been noted to produce temporary psychotic-like experiences (Luby et al., 1960; West et al., 1962). Sleep difficulties are a common prodromal feature of schizophrenia (Birchwood et al., 1989; Yung and McGorry, 1996) and even in unmedicated patients with schizophrenia there is evidence of increased sleep latency and decreased total sleep time (Chouinard et al., 2004). At a neurobiological level it has been suggested that the overactivity of dopamine D2 receptors in the striatum thought to underlie the positive symptoms of schizophrenia also enhances wakefulness (Monti and Monti, 2005).

Recent research demonstrates that paranoid thinking is much more common in the general population than previously thought (Freeman et al., 2008b). A high prevalence of insomnia has been recognised for longer. Approximately 30% of the general population experience symptoms of insomnia, with a third of this group having chronic insomnia (Ohayon, 2002; Walsh, 2004; Morin et al., 2006). This sleep disturbance is associated with anxiety and depression (Taylor et al., 2005; Breslau et al., 1996; Buckner et al., 2008); indeed, difficulties falling or staying asleep are a symptom of the diagnoses of depression, generalised anxiety disorder, and post-traumatic stress disorder (APA, 2000). In the current study the aim was to determine whether persecutory ideation and insomnia are associated. A community sample was used to obtain a range in paranoia severity and avoid the complicating issues of neuroleptic medication and high levels of inactivity. At the same time the occurrence of insomnia in a group of patients with persecutory delusions attending psychiatric services for psychosis was also examined.

## 2 Method

### 2.1 Participants

The community sample comprised 300 individuals. There were three entry criteria: aged 18 or above; able to read and write in English; and no history of treatment for severe mental illness (e.g. schizophrenia, bipolar disorder). The sample was recruited via the distribution to local postcodes of leaflets advertising research studies at King's College London. Those who responded to the leaflet were screened for the entry criteria over the telephone. Questionnaires were then completed at King's College London, by postal return or an Internet website (only available to screened individuals). The clinical group was recruited from adult services in the South London and Maudsley NHS Foundation Trust. The entry criteria were the presence of a current persecutory delusion, which met the criteria of Freeman and Garety (2000), and a clinical diagnosis of schizophrenia, schizo-affective disorder, or delusional disorder (i.e. nonaffective psychosis).

### 2.2 Measures

#### 2.2.1 Insomnia Severity Index (ISI) (Bastien et al., 2001)

The ISI is a seven-item self-report questionnaire based upon the insomnia criteria of the Diagnostic and Statistical Manual of Mental Disorders (APA, 1994). The scale assesses sleep-onset and sleep maintenance difficulties, associated distress, and interference with daily functioning. Each item is rated on a 0–4 scale. The time period is the past fortnight. Higher scores indicate the presence of symptoms of insomnia. The scale was evaluated in a sample of over two hundred individuals attending a sleep disorders clinic, and has been repeatedly used in insomnia studies (Buckner et al., 2008; Savard et al., 2005; Bernert et al., 2007). The questionnaire shows convergent validity with daily sleep diaries, significant other reports and clinician ratings. In the current study the scale showed high internal reliability (Cronbach's Alpha = .89). The guidelines for the interpretation of scores are: no clinically significant insomnia (0–7), subthreshold insomnia (8–14), clinical insomnia of moderate severity (15–21) and severe clinical insomnia (22–28).

#### 2.2.2 Sleep-50 Questionnaire (Spoormaker et al., 2005)

The nine-item insomnia subscale of the Sleep-50 Questionnaire assesses difficulties falling and staying asleep over the past month, but not interference with daily functioning. Each item is rated on a scale of 1 to 4. Higher scores indicate the presence of symptoms of insomnia. The scale was psychometrically evaluated in a sample of 400 students and 250 sleep clinic patients. In the current study the internal reliability of the scale was high (Cronbach's Alpha = .88). Clinical insomnia is indicated by a score of 19 or above.

#### 2.2.3 Green et al. Paranoid Thoughts Scale — Part B (Green et al., 2008)

The G-PTS Part B is a self-report measure of the occurrence of persecutory ideation in the past month. Each of the sixteen items (e.g. ‘I was convinced there was a conspiracy against me’, ‘Certain individuals have had it in for me’, ‘I have definitely been persecuted’) is rated on a scale from 1 to 5, and conforms to a clear definition of persecutory ideation (Freeman and Garety, 2000). The total score can range from 16 to 80. Higher scores indicate greater levels of persecutory thinking. The questionnaire has been psychometrically evaluated for use in both clinical and non-clinical populations. The internal consistency of the scale and test–retest reliability are good. Convergent validity with the Paranoia Scale (Fenigstein and Vanable, 1992) has been shown. In the current study the scale had very high internal reliability (Cronbach's Alpha = .94).

#### 2.2.4 Depression Anxiety Stress Scales (Lovibond and Lovibond, 1995)

The DASS is a 42-item instrument with three subscales measuring symptoms of depression, anxiety, and stress over the past week. Each of the subscales consists of 14 items with a 0–3 scale (0 = did not apply to me at all, 3 = applied to me very much). Higher scores indicate higher levels of emotional distress. The scale has been shown to be reliable and valid in large clinical and non-clinical populations (Brown et al., 1997; Crawford and Henry, 2003; Page et al., 2007). The anxiety and depression sub-scales were used; each showed very high internal reliability in the current study (Anxiety Cronbach's Alpha = .91, Depression Cronbach's Alpha = .96). There are no items on the scales that assess sleep difficulties.

### 2.3 Analysis

Analyses were carried out using Stata Version 10.0 (StataCorp, 2008). Visual inspection showed that the measures of persecutory ideation, anxiety and depression in the community sample all showed considerable positive skew; 46.7%, 34.3% and 31.0% had the minimum scores on the measures of paranoia, depression and anxiety respectively. These variables were therefore recoded into ordinal categories: Paranoia (16, 17–20, 21–24, 25–28, 29+), Depression (0, 1–3, 4–6, 7–9, 10–12, 13+), Anxiety (0, 1–3, 4–6, 7–9, 10+). The main analyses were ordinal logistic regressions (using the Stata ologit command) with paranoia as the dependent variable. In the first stage insomnia was the independent variable (controlling for age, sex, and working status). In the second stage depression was added as an additional independent variable. In the final stage anxiety was added to the model. This procedure was carried out separately for each of the insomnia scales. There were no missing data. The models were repeated adding the data for ethnicity and education level, which had missing data for three cases, but the results were unaltered. 95% Confidence Intervals (CI) are reported.

## 3 Results

### 3.1 Community group

The community group comprised 140 men and 160 women. The mean age was 37.7 (SD = 12.5). The reported ethnicities were: White (*n* = 216), Black-Caribbean (*n* = 16), Black African (*n* = 18), Black-Other (*n* = 6), Indian (*n* = 6), Pakistani (*n* = 1), and other (*n* = 35). One hundred and six participants were working full-time, seventy were working part-time, thirteen were retired, sixty-four were unemployed and forty-seven were students. The educational qualifications were: none (*n* = 19), GCSE (*n* = 57), AS/A-level (*n* = 41), diploma (*n* = 32), degree (*n* = 97), postgraduate degree (*n* = 53).

Scores on the measures are shown in Table 1. Based on the Insomnia Severity Index, 216 (72%) participants had no clinically significant insomnia, 62 (20.7%) participants had sub-threshold insomnia, 17 (5.7%) participants had clinical insomnia of moderate severity and 5 (1.7%) participants had severe clinical insomnia. Therefore 28% of the community sample had some degree of sleep difficulty. There was a high correlation between ISI and Sleep-50 Insomnia scores, *r* = .83, *p* < .001. Forty-six individuals (15.3%) scored above the cut-off on the Sleep-50, indicating potential clinical insomnia.

Ordinal logistic regressions examining the association of paranoia and insomnia are reported in Table 2. There is a strong association of the insomnia scales with paranoia. For the interpretation of the results it should be remembered that the odds ratios for the insomnia scales refer to one-point changes. A ten-point increase in the Insomnia Severity Index is associated with an odds ratio of 4.4 for an increase in paranoia category (1.16 raised to the power of 10). A ten-point increase on the Sleep-50 scale is associated with an odds ratio of 6.2 for an increase along the paranoia ordinal scale. 70% of those in the highest category of paranoia scores had at least sub-threshold insomnia difficulties as assessed by the ISI, whereas this was the case for only 17% of the participants without any paranoid ideation. On the Sleep-50 questionnaire, 59% in the highest paranoia category scored above the cut-off for insomnia, whereas only 8% in the lowest paranoia category scored similarly. It can be seen that when depression is added to the regression models that the relationship of insomnia to paranoia is lessened but that there is a unique contribution of each variable to predicting paranoia scores. When anxiety is added then the relationship of insomnia to paranoia is lessened substantially further, becoming statistically non-significant for the ISI.

### 3.2 Clinical group

The persecutory delusions group comprised 18 men and 12 women. The mean age was 44.2 (SD = 11.7). The reported ethnicities were: White (*n* = 16), Black-Caribbean (*n* = 3), Black African (*n* = 5), Black-Other (*n* = 3), Indian (*n* = 1), and other (*n* = 2). Twenty-two patients were unemployed, three were working part-time, three were retired, and one was a student. The educational qualifications were: none (*n* = 6), GCSE (*n* = 10), AS/A-level (*n* = 5), diploma (*n* = 6), degree (*n* = 1), postgraduate degree (*n* = 2). The diagnoses were: schizophrenia (*n* = 24), schizo-affective disorder (*n* = 4) and delusional disorder (*n* = 2). Antipsychotic medication data were converted into chlorpromazine equivalents grouped into low (0–200 mg), medium (200–400 mg) and high (≥ 400 mg); one person was not taking any medication, eleven were on a low dose, fourteen were on a medium dose and four were on a high dose. Seven patients were taking clozapine, seventeen patients were taking other atypical antipsychotics, and five people were on typical antipsychotic medication. Five patients were being prescribed two different antipsychotic drugs.

The prevalence of insomnia in the persecutory delusions group as assessed by the ISI was: 27% (*n* = 8) severe clinical insomnia, 27% (*n* = 8) clinical insomnia of moderate severity, and 30% (*n* = 9) subthreshold insomnia. Only 17% (*n* = 5) had no clinically significant insomnia. There was a high correlation between ISI and Sleep-50 scores, *r* = .78, *p* < .001. Sixty percent (*n* = 18) of the persecutory delusions group scored above the Sleep-50 insomnia cut-off. Scores did not differ by medication level on the ISI, *F*(2, 26) = .137, *p* =.872, or the Sleep-50, *F*(2,26) = .350, *p* = .708.

## 4 Discussion

The rates of sleep difficulties in the community sample were consistent with the epidemiological literature; almost 30% had symptoms of insomnia and approximately 10% were in the clinical range. But the unique focus of the study was on a potential association between sleep difficulties and paranoid thinking. The results were clear: higher levels of insomnia were associated with higher levels of persecutory thinking. Confirmation was provided by the high prevalence of insomnia in the individuals with clinical paranoia. Insomnia is most likely an overlooked problem in psychiatric services for individuals with persecutory delusions.

Sleep disturbance is already incorporated into a theoretical account of persecutory delusions (Freeman et al., 2006), but its role had not been directly tested. In this study the relationship between sleep and paranoia was largely accounted for by levels of anxiety and depression. This is unsurprising; both paranoia and sleep difficulties are closely linked with negative affect. Nevertheless the mediating routes between insomnia and paranoia require closer (and more rigorous) examination. A second plausible route is via the occurrence of perceptual anomalies. A lack of sleep may cause a puzzling internal state for the individual that, in the context of anxiety, is incorrectly attributed to external threat. Study of the mechanisms underlying insomnia in patients with persecutory delusions is also needed; sleep disturbance may be partly maintained by established insomnia-related processes such as rumination, attention to sleep-related threat, and dysfunctional beliefs about sleep (Harvey et al., 2005).

The representativeness of the samples in the current study is questionable; the community group was only a minority of the many people leafleted in the local postcodes, while the clinical group was of a small size. There was also a reliance on self-report assessments. The study focussed on a specific experience (paranoid ideation), but it could be argued that completion of a full diagnostic screening interview by the non-clinical population would have provided information of interest. However the key limitation of the study is the cross-sectional design. The causal direction of the relationship between insomnia and paranoia is unknown. It is plausible that the insomnia assessed is simply a consequence of living with paranoid fears, although a circular relationship between insomnia, anxiety and paranoia is more probable. Longitudinal studies of the relationships are clearly warranted. A priority is the evaluation of well-established insomnia interventions (e.g. Espie, 2006; Harvey et al., 2007) for individuals with delusions. These interventions will provide a stronger test of the causal relationship between insomnia and paranoia and hold the promise of enhancing the efficacy of treatments for delusional beliefs.

## Role of funding source

Funding for this study was provided by a Fellowship awarded by the Wellcome Trust to Dr. Daniel Freeman. The Wellcome Trust had no further role in study design; in the collection, analysis and interpretation of data; in the writing of the report; and in the decision to submit the paper for publication.

## Contributors

Daniel Freeman designed the study, analysed the data and wrote the paper. Katherine Pugh, Natasha Vorontsova, and Laura Southgate collected the data and commented upon the manuscript.

## Conflict of interest

There were no conflicts of interest.

## Figures and Tables

**Table 1:** Questionnaire scores

	Community group (*N* = 300)			Persecutory delusions group (*N* = 30)		
	Mean	SD	Min.–Max.	Mean	SD	Min.–Max.
Insomnia Severity Index	5.90	5.17	0–28	15.07	7.34	0–27
Sleep-50 Insomnia	13.89	4.95	9–35	23.07	8.44	11–36
GPTS-Part B (persecutory ideation)	19.94	7.85	16–73	65.27	13.49	39–80
DASS — Depression	4.42	6.99	0–42	26.63	11.17	2–41
DASS — Anxiety	3.32	5.56	0–38	21.93	10.90	4–41

**Table 2:** Ordinal logistic regressions for the community sample (*N* = 300) with paranoia as the dependent variable and controlling for age, sex and work status

	Odds ratio	*p*-value	95% CI
1. Insomnia Severity Index	1.16	< .001	1.11, 1.22
2. Insomnia Severity Index	1.09	< .001	1.04, 1.15
Depression	1.73	< .001	1.46, 2.03
3. Insomnia Severity Index	1.04	.180	.98, 1.09
Depression	1.33	.002	1.11, 1.60
Anxiety	2.22	< .001	1.67, 2.96
1. Sleep-50 Insomnia	1.20	< .001	1.14, 1.26
2. Sleep-50 Insomnia	1.13	< .001	1.07, 1.19
Depression	1.67	< .001	1.41, 1.97
3. Sleep-50 Insomnia	1.07	.024	1.01, 1.13
Depression	1.31	.004	1.09, 1.58
Anxiety	2.12	< .001	1.59, 2.82
